# pH-induced morphological changes of proteinaceous viral shells

**DOI:** 10.1038/s41598-019-41799-6

**Published:** 2019-03-29

**Authors:** D. Roshal, O. Konevtsova, A. Lošdorfer Božič, R. Podgornik, S. Rochal

**Affiliations:** 1 Physics Faculty, Southern Federal University, Rostov-on-Don, Russia; 2 Department of Theoretical Physics, Jožef Stefan Institute, SI-1000 Ljubljana, Slovenia; 3 Department of Physics, Faculty of Mathematics and Physics, University of Ljubljana, SI-1000 Ljubljana, Slovenia; 4 School of Physical Sciences and Kavli Institute for Theoretical Sciences, University of Chinese Academy of Sciences, Beijing, 100049 China; 5 CAS Key Laboratory of Soft Matter Physics, Institute of Physics, Chinese Academy of Sciences, Beijing, 100190 China

## Abstract

Changes in environmental pH can induce morphological changes in empty proteinaceous shells of bacteriophages *in vitro* that are very similar to changes occurring in viral capsids *in vivo* after encapsidation of DNA. These changes in capsid shape and size cannot be explained with a simple elastic model alone. We propose a new theoretical framework that combines the elasticity of thin icosahedral shells with the pH dependence of capsid charge distribution. Minimization of the sum of elastic and electrostatic free energies leads to equilibrium shapes of viral shells that depend on a single elastic parameter and the detailed configuration of the imbedded protein charges. Based on the *in vitro* shell reconstructions of bacteriophage HK97 we elucidate the details of how the reversible transition between Prohead II and Expansion Intermediate II states of the HK97 procapsid is induced by pH changes, as well as some other features of the bacteriophage maturation.

## Introduction

Understanding the physicochemical phenomena that occur in viruses during their maturation is extremely important, since only the mature virus particles are able to infect host cells. Formation of the majority of bacteriophages, the most common viruses on Earth, begins in an infected bacterium with a self-assembly of empty, almost spherical proteinaceous shells, called procapsids^1^. When the DNA is subsequently packed into a procapsid to form the mature virion, this represents a thermodynamically unfavorable process^2^ that can only be accomplished *via* an ATP-driven molecular motor^3^, also involving electrostatic interactions between the DNA phosphate backbone and the positively charged internal molecular motor wall^4^. After DNA has been packaged, the genome-containing capsids exhibit pronounced icosahedral faceting^5^ with symmetric capsid hexamers^6–8^. It is quite surprising then that the same morphological changes that occur in procapsids of bacteriophages during their maturation *in vivo* can be experimentally reproduced *in vitro* even in the absence of DNA^9^, solely by changing the pH of the bathing medium which strongly implicates the role of electrostatic interactions involved^10–12^.

A particularly useful experimental system for the study of the process of virus maturation and the concurrent morphological changes of its capsid is the bacteriophage HK97^13^. Its (pro)capsid assembles from 420 identical proteins and exhibits large-scale conformational changes during its maturation process^13,14^. The immature empty spherical procapsid that is formed at the very beginning of the maturation process is termed Prohead II. *In vivo* genome packaging into Prohead II results in the formation of the mature Head II, which has an increased average capsid diameter of more than 20% compared to Prohead II. This process is in addition accompanied by a thinning of the Head II protein shell and a transformation of its shape from spherical to icosahedral^7,9,15^.

Similar conformational changes to the ones that occur during the maturation process of HK97 can be achieved *in vitro* by transferring Prohead II from a neutral bathing solution with pH = 7 to an acidic one with pH = 4^16,17^. Oxidation of Prohead II results in the formation of four intermediate states that are not observed *in vivo*, termed the Expansion Intermediates (EI): EI-I, EI-II, EI-III, and EI-IV^13^. EI-I and EI-II are fairly similar in structure, have icosahedral shape, and are approximately 10% larger than Prohead II. The EI-I state, unlike EI-II, does not yet form cross-links and can therefore return, in a neutral environment, to the Prohead II state^18^. EI-III is further obtained by oxidation lasting about a week. Similarly to the mature capsid formed *in vivo*, EI-III is expanded by 20%, but has a spherical shape rather than an icosahedral one, characteristic of both preceding states, EI-I and EI-II. The last distinct *in vitro* state, EI-IV, is very similar to EI-III and differs only in the number of cross-links. After neutralizing the acidity level of the system, the spherical states EI-III and EI-IV transform back into the icosahedral Head II without any change in volume. Due to the appearance of cross-links, the final steps of the HK97 transformation are irreversible. However, the crosslink-defective K169Y mutant virus particles follow a similar maturation pathway, but the absence of cross-links allows the process to be reversible at the very last stage^6,19^.

Several *in vitro* maturation stages of HK97 can thus reversibly transform into each other due to reversible changes in the acidity of the surrounding medium. These reversible transformations manifest themselves as changes in the shape and size of the viral shell. Moreover, depending on the shell state, placing the shell from a neutral into an acidic environment can either increase or decrease the degree of its faceting. This behavior of viral shells is hard to explain merely by invoking a change in their mechanical properties, which are—for ideal, thin shells—characterized solely by its Föppl-von Kármán (FvK) number^20^. In addition, experimental results indicate that changes in the morphology of other capsids can be driven both by the changes in pH as well as salt concentration of the bathing medium^21,22^ that strongly influence the charge on the capsid proteins due to the presence of ionizable amino acid residues on the solvent-exposed surfaces of the proteins^10,11^.

In this work, we introduce a novel theoretical framework based on a coupling between electrostatic interactions, arising from point charges and specifically-oriented dipole moments on individual capsid proteins, and elastic deformations of viral shells. In this way, we are able to pinpoint how the electrostatic forces acting between polarized proteins lead to morphological changes observed in the (pro)capsid stages of the bacteriophage HK97 as a direct consequence of changes in pH. In particular, we demonstrate that both the increase in faceting as well as the capsid expansion during the reversible transition from Prohead II to EI-II can be explained in this way. Lastly, we show that our model predicts that dipole moments of capsid proteins can drive not only the expansion and faceting, but also contraction of otherwise electrostatically neutral shells, which should be of relevance also for virus-like particles and nanoshells of non-viral origin.

## Results

### Capsids as elastic, charged shells

**Figure 1:**
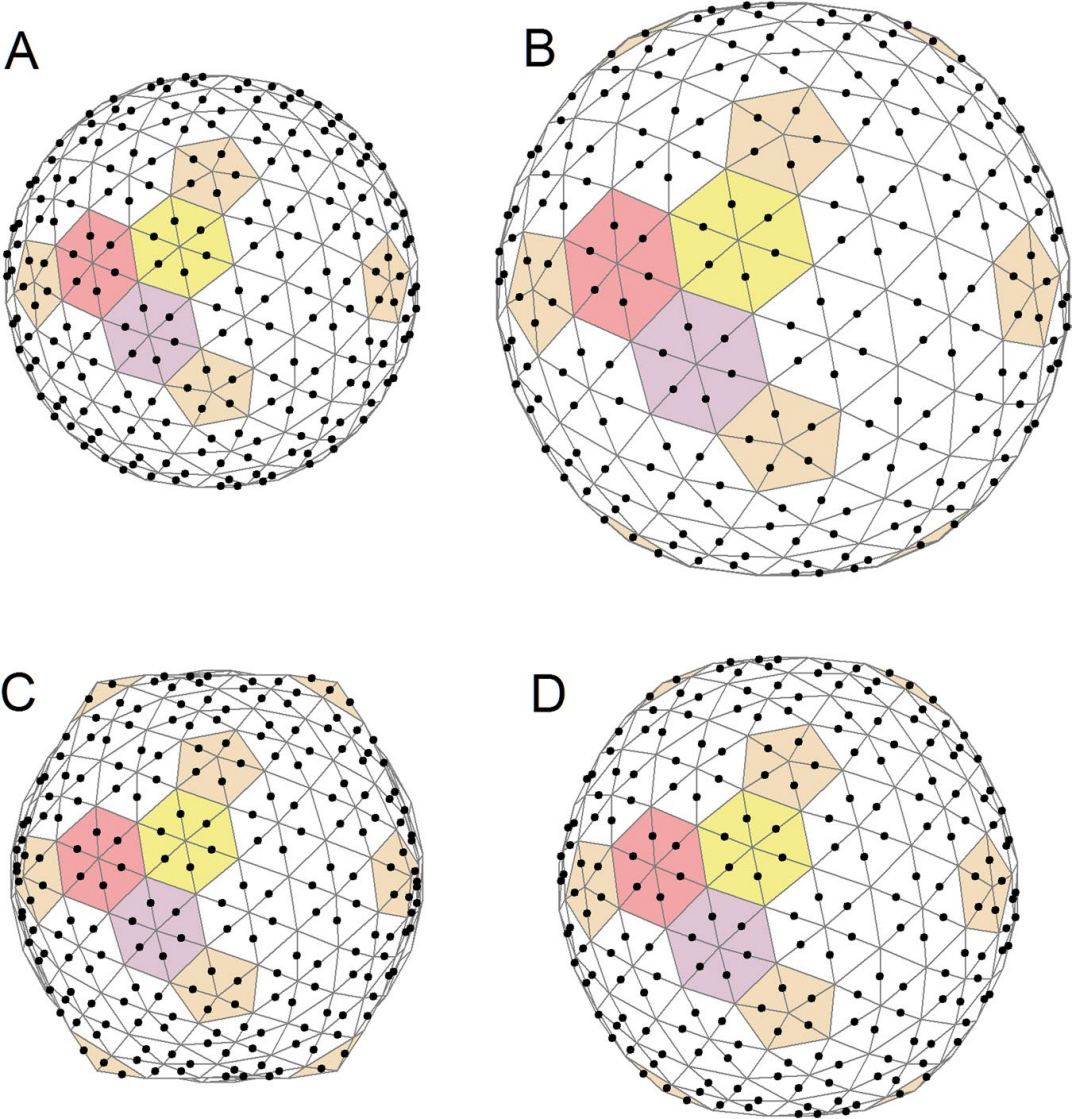
Change in shell asphericity due to addition of normalized charge. This change is determined by the shape (dependent, in turn, on the FvK number) of the initial, uncharged shell. Shells in panels (A) and (C) are uncharged (*ζ* = 0) and have different FvK numbers (120 and 600, respectively), while the ones in panels (B) and (D) have identical normalized charge (*ζ* = 0.05), with 420 equivalent point charges located at positions shown by black points. The increase in *ζ* from 0 to 0.05 for an initially almost spherical shell (A) leads to weak faceting, shown in panel (B). On the other hand, the same increase in *ζ* for an initially highly faceted structure (C) leads to a sharp reduction in its faceting (D). All the shells shown have the same bending rigidity, while the Young modulus in panel (A) is smaller than the one in panel (C), and consequently the expansion of the first shell at the same value of normalized charge is greater. Coloring of the triangular lattice drawn on the shell highlights the icosahedral symmetry and the relationship between the lattice and the CK model of capsids^28^.

About 15 years ago, Nelson *et al*.^20,23^ applied the thin-shell elasticity theory to elastic shells with spherical topology and icosahedral symmetry, consisting of identical finite elements of a regular triangular shape (as shown in Fig. 1). These shells, which can easily change their form from almost spherical to icosahedrally faceted, are often used to approximate the shape of viral capsids with different faceting^20,23–27^. In the thin-shell model^20,23^ the shell shape is controlled by a single dimensionless parameter, the Föppl-von Kármán (FvK) number $\,\gamma =\frac{Y{R}^{2}}{\kappa }$, where *Y* is the two-dimensional Young’s modulus of the shell, *κ* is its bending rigidity, and *R* is the average radius of the shell. The degree of shell faceting is characterized by the asphericity ${A}_{s}=\frac{ < {\rm{\Delta }}{R}^{2} > }{{R}^{2}}$, where the averaging takes place over all the triangulation nodes and ${\rm{\Delta }}R$ is the difference between the average value of *R* and the shell radius directed to the node. For small FvK numbers, *γ* ≤ 10^2^, shells with more than 180 triangles have essentially a spherical shape, whereas for large FvK numbers, *γ* ≥ 10^3^, they become icosahedrally faceted. (For more details, see Methods). If the capsid walls become thinner (which is often the case when the virus matures) then the FvK number grows and the shell faceting increases.

In our more complex—as well as more realistic—approach that takes into account also the electrostatic interaction between proteins, we analyze how the shape of the viral shell is controlled not only by its size and elastic properties, but in addition by the positions and magnitudes of the protein-embedded charges. We express the coordinates of these charges *via* the coordinates of the shell vertices (**R**_*i*_, **R**_*j*_, **R**_*k*_), forming *N* triangular finite elements with which the charge is associated. If the indices *t* and *q* run over the charges within one triangle and all the triangles carrying the charges, respectively, then the charge coordinates can be defined as:

1 $${{\bf{r}}}_{t}^{q}={\alpha }_{t}^{q}{{\bf{R}}}_{i}+{\beta }_{t}^{q}{{\bf{R}}}_{j}+{\gamma }_{t}^{q}{{\bf{R}}}_{k}+{\mu }_{t}^{q}R{{\boldsymbol{n}}}_{ijk},$$

where ***n***_ijk_ is the unit normal vector of a triangle. The average radius *R* in the last term makes the coefficient ${\mu }_{t}^{q}$ dimensionless, akin to the other coordinate coefficients in Eq. (1). These coefficients furthermore obey the condition ${\alpha }_{t}^{q}+{\beta }_{t}^{q}+{\gamma }_{t}^{q}=1$, implying that the charges with ${\mu }_{t}^{q}=0$ lie in the plane of the triangle. As a consequence, ${\mu }_{t}^{q}$ becomes nonzero if the shell has a finite thickness and charges can be located not only at points on the shell surface, but also above it. During the shell deformation its triangular faces also deform. Since the face strain is assumed to be linear and the charges are always attached to the same points of the shell face, the coefficients in Eq. (1) are thus fixed during the shell deformation. Note also that in the symmetry-equivalent triangles, the coordinate coefficients are the same. For example, for an icosahedral triangulation of the sphere formed by *N* = 420 triangles (the triangulation in Fig. 1) containing one charge per triangle, it is necessary to specify only 7 sets of coefficients $\{{\alpha }_{1}^{q},{\beta }_{1}^{q},{\gamma }_{1}^{q},{\mu }_{1}^{q}\},$ where *q* = 1, 2, …, 7.

If the charges attached to the shell are identical and their arrangement is fixed, then the model is controlled by only two dimensionless and non-negative scale-invariant parameters (for more details, see Methods). The first of them is the classical FvK number that characterizes the mechanical properties of the shell, while the second one, equal to the normalized charge describing the coupling between the electrostatics and elasticity, has the form:

2 $$\zeta =\frac{{Q}^{2}}{\tilde{\kappa }R},$$

where *Q* is the magnitude of the charge, $\tilde{\kappa }=2\kappa /\sqrt{3}$ (for more details, see Methods), while *R* denotes the average shell radius at *Q* = 0 (the same average radius as in Eq. (1) and in the expression for the FvK number).

### Changes in shape and size of charged elastic shells

Before we focus on the pH-induced morphological changes of capsid shells during different stages of the maturation pathway of HK97, we study the general behavior of our model in more detail. To this purpose, we place identical charges symmetrically along the edges of a triangulated sphere with *N* = 420, as shown in Fig. 1. The resulting spherical triangulation indices^28^ are *h* = 4 and *k* = 1, while the corresponding triangulation number $T={h}^{2}+{k}^{2}+hk$ equals 21. The 420 triangles of the triangulation can be further combined into 60 hexamers and 12 pentamers (see the coloring in Fig. 1). Thus, in the framework of the Caspar-Klug (CK) theory^28^, this triangulation corresponds to a model of viral capsid with ${h}_{CK}=2,{k}_{CK}=1,{T}_{CK}=7$ which are the geometric characteristics of the HK97 capsid. Furthermore, if the first vertex of triangles in Eq. (1) (with the coefficient ${\alpha }_{1}^{q}$) is chosen to be located at the capsomere centers, then for the shells shown in Fig. 1, all 7 sets of coefficients (determining the charge coordinates through the coordinates of the vertices in Eq. (1)) coincide and can be written as ${\alpha }_{1}^{q}={\beta }_{1}^{q}=\frac{1}{2};{\gamma }_{1}^{q}={\mu }_{1}^{q}=0$. Note that for each CK viral shell with a triangulation number ${T}_{CK}$ there exists also a dual spherical triangulation (with $T=3{T}_{CK}$) in which the number of triangles *N* is equal to the number of proteins (equal to $60{T}_{CK}$) in the considered shell.

Since identical charges repel, the average radius of the shell *always increases* when a shell is charged up (see Fig. 1). There is, however, no analogous universality for shell faceting that can respond to electrostatic interactions in different ways, depending on the initial asphericity (or the FvK number *γ*) of the uncharged shell. Panels A and C of Fig. 1 show two shells with zero charge, the same average radius, and asphericities equal to 1.2 × 10^−5^ and 10^−3^, respectively. Asphericity of an initially almost spherical shell (Fig. 1A) with *γ* = 120 increases to 5.9 × 10^−5^ after the shell expands by 40% due to added charge (*ζ* = 0.05; Fig. 1B). Such a growth in asphericity is mainly due to the increase in the average shell radius *R* (note that the FvK number is proportional to *R*^2^). An initially faceted shell, shown in Fig. 1C (γ = 600), expands by 15% when charge is added (*ζ* = 0.05; Fig. 1D), while in addition the expansion of the shell leads to a strong loss of asphericity (all the way to 1.5 × 10^−4^) on account of which the shell becomes visually rounder. This effect is similar to the one observed when inflating a ball, which consequently becomes rounder. In addition, we note that if the coefficients ${\mu }_{1}^{q}$ are nonzero, shell faceting becomes much more sensitive to variation in the capsid charge, a property that will turn out to be important in the discussion of HK97 procapsid states later on. Also note that with an increasing value of *ζ*, the model can lose its stability at sufficiently large values of coefficients ${\mu }_{1}^{q}$. However, in the region of the stability of the model, a gradual and continuous variation in the positions and magnitudes of the charges leads to a gradual change in the faceting and size of the shell, while a reverse variation follows the same path without hysteresis.

### pH-driven transition between Prohead II and EI-II

We can now apply our model to the specific case of the morphological transformation that occurs between Prohead II and EI-II states of the HK97 procapsid, induced by a change in bathing solution acidity. Using structural information deposited in the Protein Data Bank (PDB)^29^ and the approach detailed in ref.^10^ (see also Methods), we have calculated the centers of positive and negative charges for seven symmetry-nonequivalent types of proteins that form the structures of Prohead II (PDB:2GP1) and EI-II (PDB:3DDX), which are observed at pH = 7^7^and pH = 4^19^, respectively. The obtained positions of the centers of positive and negative charges as well as their magnitudes (listed in Table 1) rely on the assumption that the dielectric constant of the medium is independent of the pH value, and that the same elastic shell (*N* = 420, *γ* = 250) is used to model both charged procapsid states.

**Table 1:** Positions and magnitudes of the centers of positive and negative protein charges of the Prohead II (pH = 7) and EI-II (pH = 4) states of HK97 procapsid (obtained from PDB:2GP1 and PDB:3DDX, respectively).

Protein	Prohead II (pH = 7)						Expansion Intermediate II (pH = 4)					
	*Q*_+_ = 23.393424 [e_0_]			*Q*_−_ = −29.977607 [e_0_]			*Q*_+_ = 28.963847 [e_0_]			*Q*_−_ = −18.776508 [e_0_]		
	x [nm]	y [nm]	z [nm]	x [nm]	y [nm]	z [nm]	x [nm]	y [nm]	z [nm]	x [nm]	y [nm]	z [nm]
A	5.51	8.94	20.4	5.16	8.75	20.73	5.52	10.24	23.84	5.56	10.06	23.7
B	8.49	9.12	19.17	8.17	9.49	19.33	9.24	10.16	22.16	9.17	9.97	22.16
C	9.86	5.8	19.35	9.99	6.28	19.3	11.35	6.7	21.97	11.12	6.7	21.96
D	7.97	2.92	20.66	8.44	3.13	20.56	9.58	3.33	23.25	9.45	3.48	23.21
E	5.08	2.75	21.98	5.46	2.42	22.02	5.78	3.34	24.65	5.85	3.5	24.49
F	3.77	6.08	21.8	3.72	5.62	22.01	3.91	6.95	25.1	4.1	6.92	24.91
G	1.53	10.40	21.30	1.97	10.66	21.24	1.72	12.43	24.75	1.66	12.54	24.54

The following notation is used: type G proteins form pentamers, and the remaining protein types form hexamers. Type A proteins are in contact with those of type G; proteins A–F are located counter-clockwise in the hexamers.

**Figure 2:**
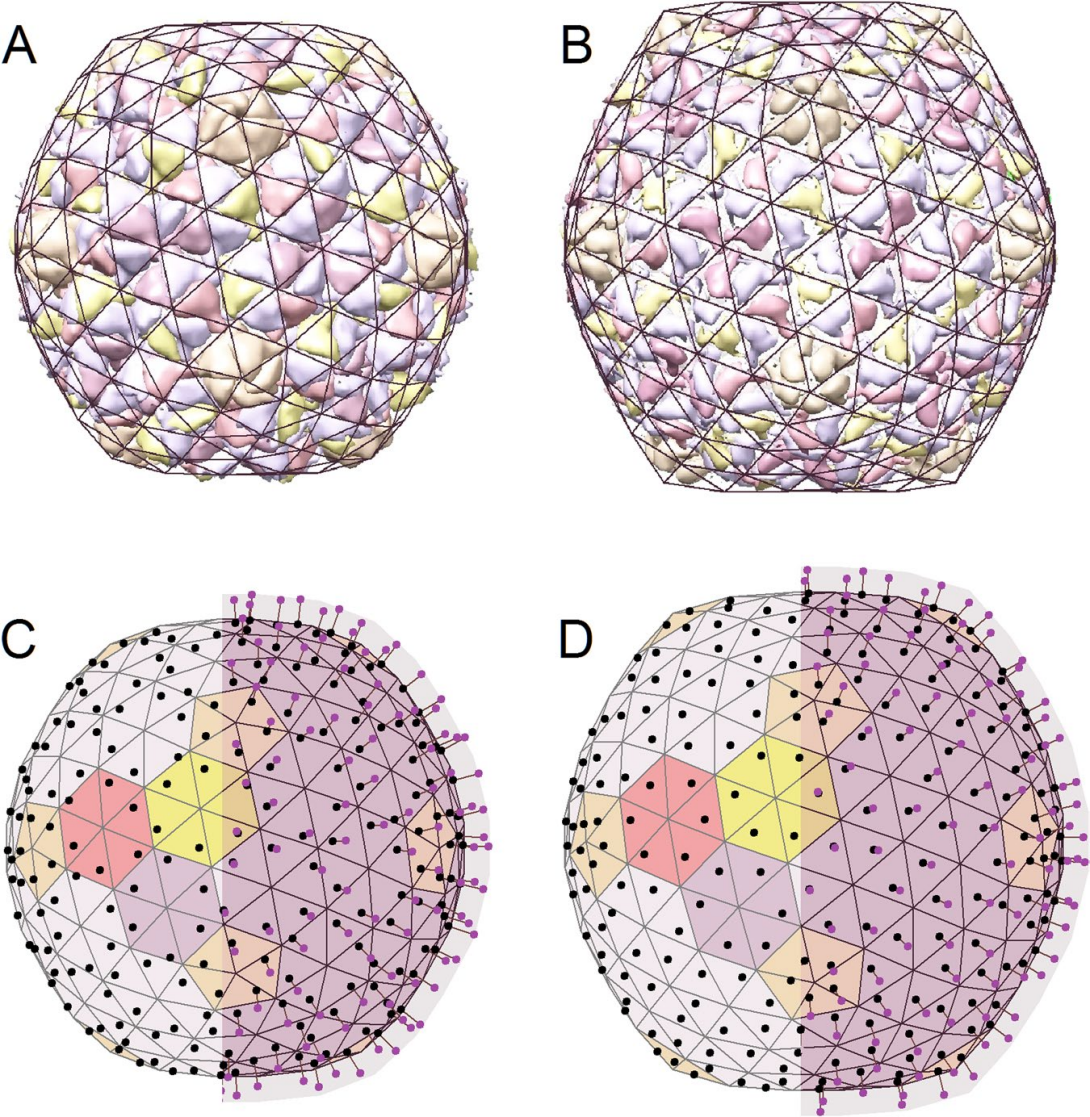
Reversible transformation from Prohead II to EI-II state of the HK97 procapsid due to a change in acidity from pH = 7 to pH = 4. Panels (A) and (B) demonstrate the two procapsid structures (PDB:2GP1 and PDB:3DDX) drawn using UCSF Chimera^35^ and superimposed with their icosahedral triangulations (uncharged phantom shells) characterized by *A*_*s*_ = 5.5 × 10^−4^ and *A*_*s*_ = 10^−3^, respectively. Upon the change in acidity, both positions and magnitudes of charges on the procapsid proteins change concurrently. In panels (C) and (D), these charges are shown as small balls fixed at different positions above the elastic shells, which have identical mechanical properties (*γ* = 250). The left halves of these two panels show only the charge projections above the shell surfaces. The pH-induced variation in protein charges changes both the shape and the size of the shells (panels (C) and (D)), making them similar to experimentally-observed procapsid structures (panels (A) and (B)). In our coarse grained approach, the change in asphericity between the two model shells (panels (C) and (D)) is slightly smaller than the one observed experimentally between the procapsid states (panels (A) and (B)).

To apply our model, the charge coordinates from Table 1 have to be converted into sets of coefficients $\{{\alpha }_{t}^{q},{\beta }_{t}^{q},{\gamma }_{t}^{q},{\mu }_{t}^{q}\}$ [see Eq. (1)] for both procapsid structural states, Prohead II and EI-II. For this recalculation, we need the values of **R**_*i*_ vectors which can be obtained by triangulating the outer surface of the procapsid states under consideration. The desired triangulations, which allow us to replace the real protein structure with a zero-thickness phantom shell of the same asphericity, can be constructed in several different ways. We obtain the triangulations by simply varying the FvK numbers at zero charge. The average radii and the asphericities (5.5 × 10^−4^ and 10^−3^ for Prohead II and EI-II, respectively) of the resulting phantom shells are fitted in such a way that they optimally correspond to the surfaces of the procapsid states in question (Fig. 2A,B).

After the phantom shells are obtained, we use Eq. (1) and the data in Table 1 to get the coordinate coefficients of the model. Their values—especially those of ${\mu }_{t}^{q}$—depend on the magnitude of *R* [see Eq. (1)], which in this model does not necessarily coincide with the average external radius of the capsid. If we calculate *R* as the average distance *R*_m_ from the capsid center to the mass centers of proteins and stick to the assumption that the elastic properties during the pH-driven transition remain unchanged, then it is not possible to fit the change in shape between the Prohead II and EI-II states. The most successful fit is obtained if the value of *R* is about 10% less than *R*_m_; thus, the charges are effectively positioned above the elastic shell, and in this way the nonzero thickness of the capsid wall is also taken into account even in the thin-shell elastic model. This fitting procedure, which allows us to express the coordinates of charges (given in Table 1) in the local coordinate systems of triangular finite elements, thus supports our assumption that during the reversible pH-driven transition any change in the elastic properties of the shell is inessential and the shell varies its shape and size principally due to the shift of charges’ positions and the change in their magnitudes.

While we have initially calculated the change between the Prohead II and the EI-II states by placing the centers of positive and negative charges at different positions above the same elastic shell, it turns out that in this particular case, the two-charge (or *bipolar*) approximation can be simplified and reduced to an equivalent *monopolar* one. Inspecting Table 1, we discern that the distance between the centers of positive and negative charge in all proteins is much smaller than the thickness of the proteinaceous layer itself, while the total protein charge remains significant. Therefore, we can preserve the accuracy of the model by positioning an equivalent total charge *Q*_*tot*_ = *Q*_+_ + *Q*_−_ at the *center of charge*, **r**_*tot*_ = (**r**_+_*Q*_+_ + **r**_−_*Q*_−_*)/Q*_*tot*_, where **r**_+_ and **r**_−_ are the coordinates of positive and negative charges, respectively. This monopolar model still explains well the procapsid transformation in question, and the equivalence of both models is ensured by the fact that the dipolar moment vanishes if it is calculated with respect to **r**_*tot*_.

Panels C and D of Fig. 2 show how the shape of the model Prohead II state transforms to the model EI-II state due to the pH-driven change of the positions and magnitudes of total charges on the two shells (as presented in Table 1). These two states have the same mechanical properties (*γ* = 250) but display quite different shapes due to different charge arrangements and electrostatic energies. The Prohead II state is characterized by *A*_*s*_ = 1.6 × 10^−4^ and *ζ*_*c*_ = 0.43, while the EI-II state is characterized by *A*_*s*_ = 3.5 × 10^−4^ and *ζ*_*d*_ = 1.04. The ratio $\frac{{\zeta }_{{\rm{c}}}}{{\zeta }_{d}}$ is equal to the squared ratio of total charge of the two states [see Eq. (2)], and this difference in total charge explains the increased average radius of the EI-II state (by about 9.5%) compared to the Prohead II. Interestingly, in the Prohead II state the presence of charge leads mainly to an expansion of the shell (with respect to the uncharged phantom shell with the same mechanical properties), while in the EI-II state, the different positions of charge centers result also in an increase of the shell asphericity. Additionally, we note that the hexamers in Prohead II are flattened (Fig. 2A), which is reflected also in the positions of the charge centers, shown in Fig. 2C. After the transition to the EI-II state (Fig. 2B), the hexamers become more regular, while a weak asymmetry still exists at the positions of charge centers (Fig. 2D).

### pH-driven transition between Balloon particle and Head II

The same framework can also be used to briefly discuss the transition that occurs at the last stage of the HK97 capsid formation between the Balloon particle (EI-III/EI-IV) and the Head II state. During prolonged oxidation, which results in the transformation from EI-II to EI-III, the mechanical properties of the procapsid shell change. Because of the reduction of the capsid wall thickness *d*, both the Young’s modulus (which is proportional to *d* for a homogeneous material) and the bending rigidity (proportional to *d*^3^) simultaneously decrease. Within the confines of the thin shell elasticity^20,23^, under such conditions the resulting FvK number should increase and the shell should become more faceted. However, despite the increase in the FvK number, the observed Balloon state becomes more spherical. This can be straightforwardly understood within our framework as the (uncharged) shell with smaller elastic constants has mechanical properties similar to those of an actual balloon, while the inclusion of charges leads to its expansion, just as compressed air inside the balloon makes the balloon rounder^12^.

We have also attempted to explain the slight increase in the faceting observed in the Head II state (PDB:2FT1) when compared to the Balloon state EI-IV (PDB:2FTE) using the bipolar approximation, described above, and under the assumption that the mechanical properties of the two states coincide. In this case, however, our model could not reproduce the experimentally observed increase in faceting, possibly due to several reasons. It could be that in the last procapsid state of HK97, the FvK number increases due to the formation of cross-links between proteins, resulting in a change in the elastic properties of the shell, not accounted for in our model. While a similar reversible transformation between two states (in the absence of cross-links) is observed in the genetically defective K169Y virus particles, the analogy with the defective shell may not be entirely correct, since our electrostatic interactions were implemented specifically for the EI-IV state of HK97 and not for the defective particle. It is possible that in order to study the transformation between the EI-IV and Head II states of the normal and defective capsids, quadrupole terms or even additional details of the model need to be taken into account in the calculation of the electrostatic energy of the shell.

## Discussion

Capsid proteins in all the HK97 (pro)capsid states are charged, in both neutral as well as acidic medium. While their particular charge configurations show that the centers of positive and negative charges are close enough so that the charge on the proteins can be considered as monopolar, we comment here briefly on how protein dipole moments can in general nevertheless affect the shape and size of an overall electrostatically neutral icosahedral shell.

First of all, it is important to note that contrary to the monopolar interactions, the energy of the dipole-dipole interaction, being dependent on the dipoles’ orientation, can in general change sign. Consequently, the shell can both expand as well as contract, unlike in the case of the monopole-monopole interaction, where the shell can only expand due to the electrostatic repulsion between identical charges. Nevertheless, radial dipoles always lead to an expansion of the shell and, depending on their positions, can substantially increase the faceting of weakly-faceted shells. Radial dipoles, if placed directly on the surface of a weakly-faceted shell with *A*_*s*_ = 1.6 × 10^−4^ (the asphericity of the structure in Fig. 2C) at the same positions as the charges in Fig. 1, induce only a 10% inflation of the shell (due to dipole-dipole repulsion) but can increase its asphericity by as much as 6 times. Similar asphericity of the procapsid surface with an icosahedral triangulation is shown in Fig. 2B.

On the other hand, tangential dipoles can affect the shape and size of the shell in fundamentally different ways, depending on their positions and orientation. A full discussion of this issue goes clearly beyond the scope of this paper and will be published elsewhere, so we only briefly note a few of the most interesting findings. First of all, for certain orientations the tangential dipoles can suppress faceting without inducing any concurrent change in the shell volume, in remarkable contrast to the cases shown in panels C and D of Fig. 1. Second, it is also interesting to note that shells in a model with fixed dipoles become less stable and can collapse as the dipole moment increases, while immediately before the collapse, it is sometimes possible to observe something akin to a *crumpling* of the shell surface.

The model proposed in this work is essentially discrete, since point charges (or dipoles) are located on the shell consisting of finite elements, each of which corresponds to a single protein. On the one hand, the discreteness and relative simplicity of the model allow us to substantially increase the rate of the numerical calculations aimed at shell energy minimization, but, on the other hand, these factors also limit our approach. If necessary, the level of detail in the model can be increased, for example, by replacing one protein with several elastic finite elements corresponding to protein subunits. Namely, spherical triangulation with *T* = 21 and 420 triangles is not the only one suitable for modeling the HK97 capsid. Another triangulation with *T* number a multiple of 21 could be used, and consequently, one capsid protein would correspond not to a single triangle (as in Fig. 1), but to *T*/21 triangles of the tessellation. However, in this case the electrostatic part of the model would have to be upgraded to a similar level of detail and the electrostatic characteristics of the subunits utilized would need to be taken into account.

Notwithstanding, one can ask whether it is reasonable to increase the number of finite elastic elements and leave the electrostatic part unchanged? Or, more generally, is it reasonable to consider the model with a continuous mechanical part and discrete distributions of charges or dipoles? We analyzed this situation and obtained a rather negative answer. First, the relatively large electrostatic forces applied to relatively small finite elements of the elastic shell easily lead to instabilities in the system or to highly inhomogeneous large deformations of the shell around elements carrying charges or dipoles. Such unreal deformations are typical of shells with tangential dipoles, which induce at the points of their attachment mainly tangential electrostatic forces.

The range of validity of our model is thus set by its coarse-grained nature in general, but more specifically also by the fact that we have not considered some of the salient features of the electrostatic interactions in the context of proteinaceous shells^30,31^, such as dielectric inhomogeneities, ionic specificity and finite electrolyte screening. While inclusion of these effects is of course in principle desirable, it would introduce new parameters and scaling functions which would make the analysis unnecessarily involved.

In conclusion, we report a new coarse-grained model of electrostatic-driven transformations of viral shell morphology due to variations in the pH level of the bathing medium. We show that such pH variations can lead to a significant change in the configuration of charges imbedded in the proteinaceous shells and, consequently, can affect their faceting and size. The charge configurations were calculated directly from the available detailed structural data for capsid states at different solution acidities, while the mechanical part of the model was based on the finite element approach to the capsid elasticity. We have applied this framework to the HK97 procapsid, which is a well-known model of capsid maturation that has been studied for a long time. As is well established for this case, the procapsid reconstruction *in vitro* (due to the pH decrease) is similar to that occurring during the maturation process *in vivo*. Our model, reproducing well the procapsid maturation *in vitro*, could be useful also for elucidating the design and development of antiviral strategies, since only the mature viruses can infect the host cells. In addition, the results obtained in our work can be helpful to study how changes in acidity and salt concentration affect the self-assembly of various protein nanostructures and possibly even more complex nano-objects, including various composite carbon nanomaterials with adsorbed protein or peptide molecules, since the adsorption in this case can be controlled by the pH level of environment (see some examples in^32^).

## Methods

### Coupling electrostatics and elasticity

One of the traditional ways to create a simple model of a viral shell is triangulation—treating the capsid as a system of nodes connected by lines with their nearest neighbors^20,24,28^. In this way, the (infinitely thin) spherical shell is divided into triangles, and their vertices—which are simultaneously the vertices of the polyhedron obtained from the shell—represent groups of 5 or 6 proteins, the so-called *capsomeres*. Such an approach actually forms the basis of the theory of elastic shells^20,23^, describing the onset of faceting in icosahedral capsids. In the continuum limit, the faceting of such shells was shown to be controlled by the FvK number, while the average shell radius *R* is practically independent of the degree of faceting.

Alongside the continuum model, the theory of Nelson *et al*.^20,23^ also provides a discrete model with an equivalent behavior. The discrete mesoscopic elastic free energy of a triangulated shell can be written as:

3 $${E}_{el}=\sum _{i=1}^{n}[\frac{\mathop{\kappa }\limits^{ \sim }{{\alpha }_{i}}^{2}}{2}+\frac{k{({l}_{i}-{l}_{0})}^{2}}{2}],$$

where the sum runs over all *n* edges of the triangulation, whose vertices are the capsomeres. In Eq. (3), *k* is the rigidity of the capsomere bonds located along the triangulation edges, *l*_0_ is the equilibrium length of these bonds, $\tilde{\kappa }$ is the microscopic bending rigidity, *l*_*i*_ is the nonequilibrium length of the i-th link, and *α*_*i*_ is the angle between the normal vectors of two triangles which have the i-th edge as their common side. The first term in the discrete elastic energy describes the contribution of the shell curvature, while the second term reflects the elastic extension of the bonds.

Since the area of the sphere is approximately equal to the area of the *N* triangles, it follows that $\frac{\sqrt{3}N{{l}_{0}}^{2}}{4}=4\pi {R}^{2}$. By using the connection between the elastic constants of the continuum and discrete models, $Y=\frac{2}{\sqrt{3}}k$ and $\kappa =\frac{\sqrt{3}}{2}\tilde{\kappa }$^20,33^, the FvK number of the discrete model turns out to be equal to

4 $$\gamma =\frac{kN{{l}_{0}}^{2}}{4\pi \tilde{\kappa }\sqrt{3}}.$$

**Figure 3:**
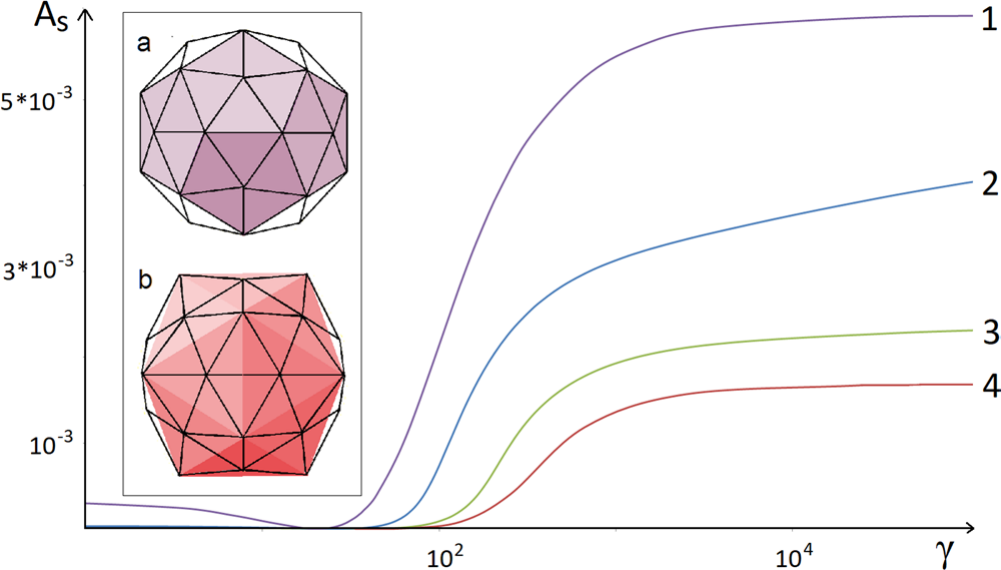
Asphericity as a function of the FvK number for several icosahedral shells. The curves labeled 1–4 correspond to shells with the number of triangles equal to *N* = 60, 180, 420, and 2160, respectively. To the right side of the region where the function *A*_*s*_(*γ*) increases rapidly (large values of the FvK number), all shell shapes become significantly icosahedral. For the case of *N* = 60 (violet curve 1), the inset shows two shells that arise in the limits *γ* → 0 (**a**) and *γ* → ∞ (**b**). These states correspond to weak dodecahedral and strong icosahedral faceting of the sphere, respectively, and the most protruding vertices of the shells are then simultaneously the vertices of either dodecahedron (violet) or icosahedron (red).

Nelson *et al*.^20,23^ considered numerically the faceting of very large shells, corresponding to the limit *N* → ∞. In order to demonstrate how the discrete model tends to the continuum limit, we show in Fig. 3 the dependence of asphericity on the FvK number for shells with *N* = 60, 180, 420 and 2160. According to the results of both *Nelson et al*.^20,23^ as well as our own, the case with *N* = 2160 already corresponds to the continuum limit. The next closest curve to the continuum limit is the triangulation consisting of 420 triangles (corresponding to the number of proteins in the HK97 capsid). Figure 3 in addition reveals a heretofore unacknowledged feature: in fact, in the limit *γ* → 0 the asphericity of discrete shells increases. This is especially noticeable in the case of a shell composed of only 60 triangles. Moreover, the asphericity in the *γ* → 0 limit does not possess an icosahedral but rather a dodecahedral character, and the shell radii lying on the 5-fold axes are smaller than those lying on the 3-fold ones (see also the discussion of dodecahedral viral shells in ref.^34^).

In our approach we related the triangular faces of the shell with individual proteins, rather than associating the shell vertices with capsomers^20,23^. What is more, we have extended the model by positioning either protein charges or dipoles onto the faces of the triangulated shell. Obviously, this extension leads to the appearance of additional electrostatic forces and their moments applied to the shell.

In the standard elastic model^20,23^ the shell shape is determined by the minimization of its elastic free energy with respect to the coordinates of the shell vertices **R**_i_. In our variant of the model, we retain the same approach and express the coordinates of the charges through the coordinates of the vertices (**R**_*i*_, **R**_*j*_, **R**_*k*_) forming the triangles to which the charge is attached (see Eq. (1) and its explanation in the text). During the free energy minimization, the coefficients in Eq. (1) are then considered as fixed. Therefore, the electrostatic energy, like the mechanical one, is ultimately a function of the shell vertices **R**_i_.

For the electrostatic part of the free energy, taking into the account the interactions between charges positioned on the shell, we assume the simple form:

5 $${E}_{Q}=\sum _{i > j}\frac{{Q}_{i}{Q}_{j}}{{r}_{ij}},$$

where the sum runs over all charges *Q*_*i*_, excluding the charges within one triangle (if index *t* in Eq. (1) can take several values). The charges are renormalized to take into account the dielectric constant, and the denominator *r*_*ij*_ is the distance between the *i*-th and the *j*-th charge. Note that the symmetry-equivalent charges should be identical. In Eq. (5), we have neglected the Debye-Hückel type of electrolyte screening, implying that our calculation is quantitative only in the case of very dilute electrolytes, but the screening and the dielectric inhomogeneities implied by the finite thickness of the proteinaceous shell to a large extent compensate one another. A more realistic approach, based on the screened electrostatic potential and dielectric inhomogeneities^30,31^, would entail additional scaling parameters and functions, thus becoming cumbersome and difficult to follow.

The first step in the analysis of our model is the observation that both the total energy, which is the sum of Eqs (3) and (5), and consequently the shell asphericity are invariant with respect to the following simultaneous rescaling of the model parameters:

6 $${{\bf{R}}}_{i}\to \zeta {{\bf{R}}}_{i},R\to \zeta R,{r}_{ij}\to \zeta {r}_{ij},{l}_{0}\to \zeta {l}_{0},\tilde{\kappa }\to \tilde{\kappa }{\zeta }^{0},k\to k{\zeta }^{-2},{Q}_{i}\to {Q}_{i}{\zeta }^{\frac{1}{2}},$$

where *ζ* is an arbitrary change of the shell size. Therefore, we can introduce a scale-invariant shell energy $\frac{{E}_{tot}}{\tilde{\kappa }}$ so that in the simplest case where all the charges on the shell are identical $({Q}_{i}=Q)$ our model is characterized by only two dimensionless, non-negative, scale-invariant parameters. The first of them is again the FvK number, characterizing the size and elastic properties of the shell, while the second one, describing the electro-mechanical coupling, is given by Eq. (2). In a more general case the number of electro-mechanical coefficients becomes equal to the number of non-identical charges.

### Determining charge on capsid proteins

The electrostatic part of our model, introduced above, requires the knowledge of the positions and magnitudes of charges on the capsid shell. While different levels of detail are available, we have decided here to represent the charge on the capsid by calculating the centers of positive and negative charges on individual capsid proteins. In this way, the computational complexity of our model is still manageable, while on the other hand we efficiently dealt with both the electrostatic monopole and dipole moments of the capsid proteins.

In order to determine the positions and magnitudes of the centers of positive and negative charges on the capsid proteins, we have followed the general procedure outlined in detail in ref.^10^. We have extracted the positions of ionizable amino acids (aspartic and glutamic acid, tyrosine, arginine, histidine, and lysine) belonging to each different type of capsid protein from structures deposited in the Protein Data Bank^29^. The degree of dissociation and the partial charge on the amino acids at a given pH value were then obtained by virtue of the Henderson-Hasselbalch equation, where we have used bulk acid-base dissociation values (*pK*_*a*_) for each amino acid type. Afterwards, we have separately determined the positions of charge centers of negatively and positively charged amino acids and their magnitudes for individual capsid proteins. While this approach can be again extended to include more details (for instance, by including site-site interactions in obtaining the *pK*_*a*_ values or charge regulation due to local electrostatic potential—see ref.^10^ for details), this would again increase the computational complexity of the model, and thus we have decided to omit them.

## Data Availability

All data needed to evaluate the conclusions of the paper are present in the paper. Additional data related to this paper may be requested from the authors.
